# Interferon Gamma and Sonic Hedgehog Signaling Are Required to Dysregulate Murine Neural Stem/Precursor Cells

**DOI:** 10.1371/journal.pone.0043338

**Published:** 2012-08-29

**Authors:** Janine Walter, Hans-Peter Hartung, Marcel Dihné

**Affiliations:** 1 Department of Neurology, Heinrich-Heine-University, Düsseldorf, North Rhine-Westphalia, Germany; 2 Department of Neurology and Epileptology, Hertie Institute for Clinical Brain Research, Eberhard-Karls-University, Tübingen, Baden-Württemberg, Germany; Universitätsklinikum Carl Gustav Carus an der Technischen Universität Dresden, Germany

## Abstract

**Background:**

The pro-inflammatory cytokine interferon gamma (IFNγ), a key player in various neurological diseases, was recently shown to induce a dysregulated phenotype in neural stem/precursor cells (NSPCs) that is characterized by the simultaneous expression of glial and neuronal markers and irregular electrophysiological properties. Thus far, the mechanisms of this phenomenon have remained unclear.

**Methodology/Principal Findings:**

To determine if binding of the signal transducers and activators of transcription (Stat 1) to the sonic hedgehog (SHH) promoter is important for this phenomenon to occur, chromatin immunoprecipitation and pharmacological inhibition studies were performed. We report here that the activation of both the Stat 1 and SHH pathways is necessary to elicit the dysregulated phenotype.

**Conclusions/Significance:**

Thus, blocking these pathways might preserve functional differentiation of NSPCs under inflammatory conditions leading to more effective regeneration.

## Introduction

The pro-inflammatory cytokine IFNγ is mainly produced by cytotoxic CD8^+^ T-cells, natural killer cells [1], astrocytes, fibroblasts and endothelial cells [2], [3], [4] under normal or pathological conditions after stroke, cerebral traumata or in the course of inflammatory brain diseases [5]. As previously reported, IFNγ affects murine NSPCs *in vitro* leading to a dysregulated phenotype [6]. This phenotype is characterized by reduced proliferative activity and a synchronous up-regulation of mature neuronal and glial markers also in the presence of growth factors. The IFNγ-induced phenotype bears electrophysiological properties that are indiscernible from undifferentiated NSPCs. The mechanisms involved in IFNγ-induced NSPC dysregulation are unknown. Up-regulation of Stat 1 after IFNγ exposure suggested one of the common down-stream pathways of IFNγ to be involved in NSPC dysregulation. Interestingly, also SHH was considerably up-regulated pointing to a possible crosstalk of IFNγ signaling and SHH production during formation of the dysregulated NSPC phenotype. Similar mechanisms were observed during the differentiation of granular neuron precursor cells of postnatal mice [7] or primary mouse and human pre-adipocytes [8] under IFNγ influence.

## Results

### Genotypic and Phenotypic Dysregulation of NSPCs and Effects of SHH Antagonism

**Figure 1:**
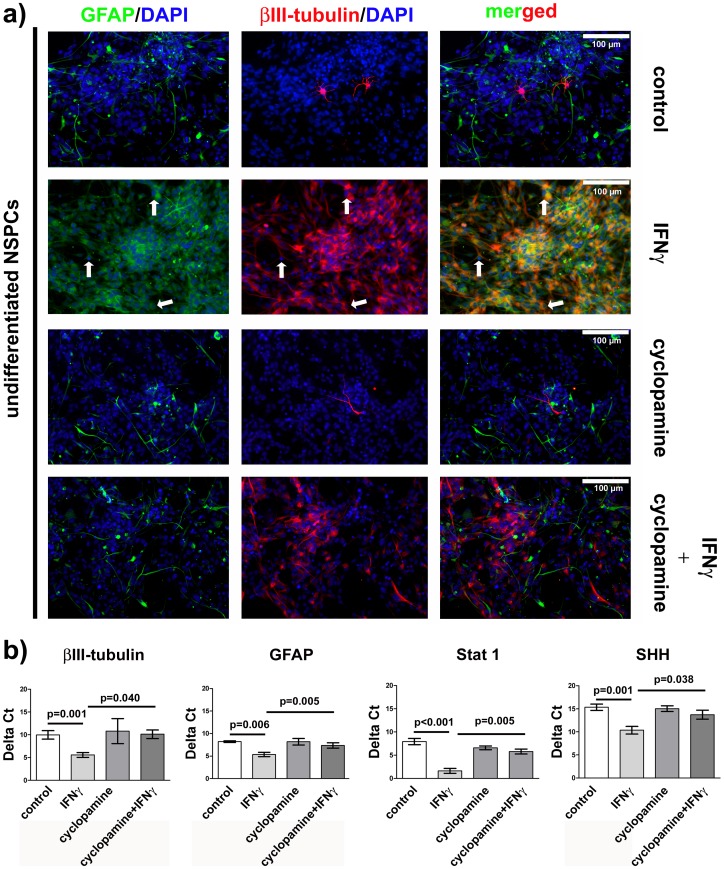
Effects of IFN γ **in NSPCs are blocked by cyclopamine.** In Figure a: Photomicrographs of undifferentiated murine NSPCs are given. The left panel shows an immunocytochemical staining for GFAP, the middle panel for βIII-tubulin and the right panel shows both. The first row represents the control, and the second row represents the IFNγ-treated cells. The third row shows cells that are treated with cyclopamine alone, and the fourth row shows cells treated with both substances. In Figure b: The results of the real-time quantitative PCR experiments for GFAP, βIII-tubulin, Stat 1 and SHH gene expression are depicted. Delta CT values are means +/− standard error of mean (SEM). Independent experiments were repeated for three times in triplicate.

To verify if SHH signaling is involved in generating the IFNγ-induced phenotype in NSPCs, we antagonized SHH signaling with cyclopamine during IFNγ exposure. Cyclopamine is known to inhibit SHH signaling due to binding, inactivation and change in protein conformation of smoothened [9]. Smoothened is a seven-pass membrane protein and G Protein coupled receptor that regulates the translocation of Gli transcription factor to the nucleus [9]. In a first set of experiments, we verified the induction of the dysregulated GFAP^+^/βIII-tubulin^+^ phenotype by IFNγ treatment of NSPCs under the influence of growth factors. As previously reported, we could reliably induce the GFAP^+^/βIII-tubulin^+^ phenotype by 1000 U/ml IFNγ (Figure 1a). Also, on mRNA level we demonstrated an up-regulation of both, GFAP and βIII-tubulin after IFNγ exposure (Figure 1b). We then inhibited the SHH pathway during IFNγ-induced dysregulation. For this purpose, we simultaneously applied cyclopamine and IFNγ. And indeed, cyclopamine nearly completely prevented the generation of GFAP^+^/βIII-tubulin^+^ cells. These findings were confirmed on protein and on mRNA level by means of immunocytochemistry and real-time quantitative PCR (Figure 1a+b). To investigate effects of SHH antagonism on proliferating, non-dysregulated NSPCs, we also applied cyclopamine without IFNγ. We found no significant differences in the expression of βIII-tubulin or GFAP in the non-treated control or the cyclopamine-treated group (Figure 1a+b).

We monitored the expression levels of Stat 1 and SHH in all 4 experimental groups by real-time quantitative PCR, since we postulate a crosstalk of IFNγ signaling and SHH pathway, probably mediated by phosphorylated Stat 1 leading to the establishment of the dysregulated phenotype of NSPCs. We found Stat 1 and SHH to be up-regulated after IFNγ exposure in comparison to the control group. Cyclopamine inhibited this IFNγ-induced up-regulation and no significant changes in Stat 1 and SHH expression in comparison to control were observed when cultures were treated with cyclopamine alone (Figure 1b).

### Gen-expression Levels of SHH and Stat 1 and Population Size of NSPCs Correlate to the Concentration of IFNγ

**Figure 2:**
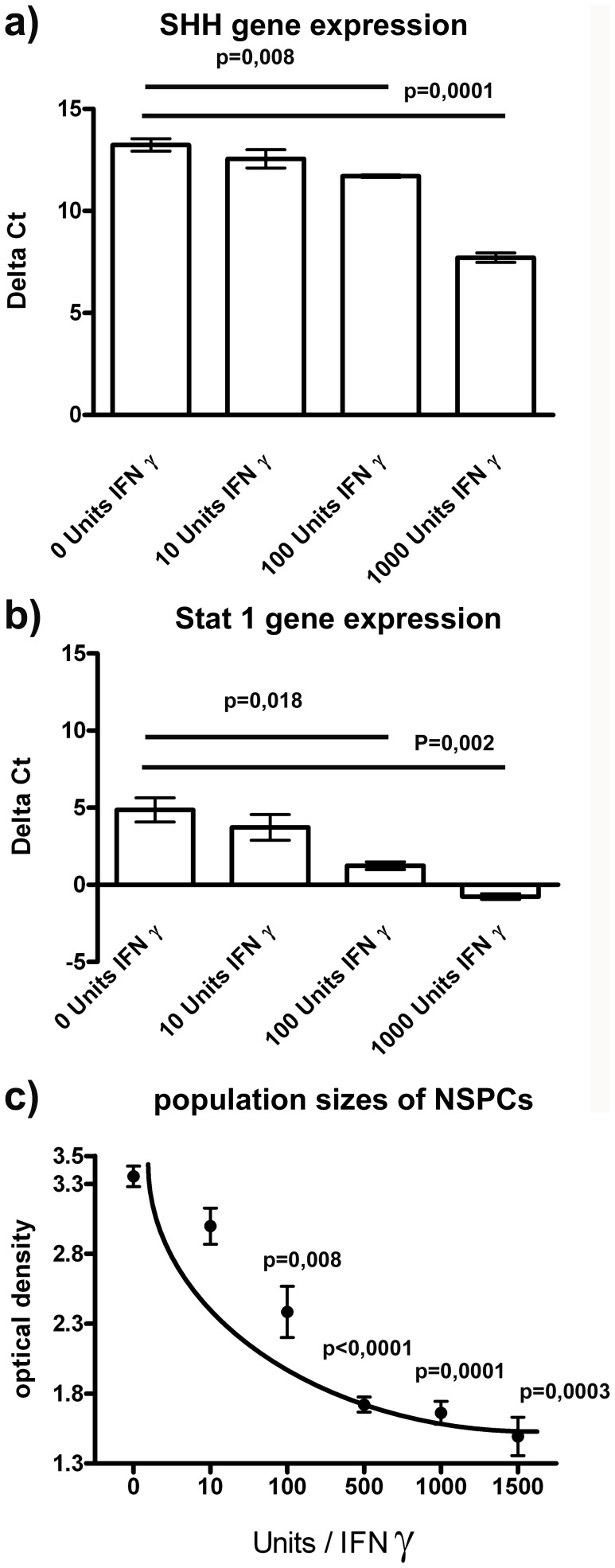
The effects of IFN γ **treatment in NSPCs are dose-dependent.** In Figure 2 a, b, c: Gene expression of SHH and Stat 1 or the population sizes of the NSPCs are shown in relation to illustrated concentrations of IFNγ. Values are means +/− standard error of mean (SEM). Independent experiments were repeated for three times in triplicate. For concentration dependent gene expression and proliferation studies, NSPCs were seeded at an equal density at day 0, than incubated with different amounts of IFNγ as indicated in the figure. The experimental read out took place three days after the start of the experiment.

To detect a possible concentration threshold from where IFNγ induces SHH and/or Stat 1 up-regulation, we performed experiments with different concentrations of IFNγ. We found a significant up-regulation of SHH and Stat 1 at an IFNγ concentration of 100 Units per ml and higher (Figure 2 a+b). We then investigated the population size of undifferentiated NSPCs under the influence of different concentrations of IFNγ since we speculate, that the above mentioned concentrations of 100 Units per ml or higher will also influence their proliferation. Undifferentiated NSPC populations treated for 72 hours with the indicated concentrations of IFNγ showed a significant decrease in optical density, which represents the measuring value in the MTT assay indicative for a reduced population size due to reduced proliferation or induced apoptosis. As the MTT assay measures metabolic activity, a bias towards lower values in differentiated cultures after IFNγ-treatment cannot completely be excluded. Again, these findings were significant with concentrations of 100 Units per ml or higher (Figure 2c).

### Administration of Recombinant SHH alone does not Lead to a Dysregulated Phenotype in NSPCs

**Figure 3:**
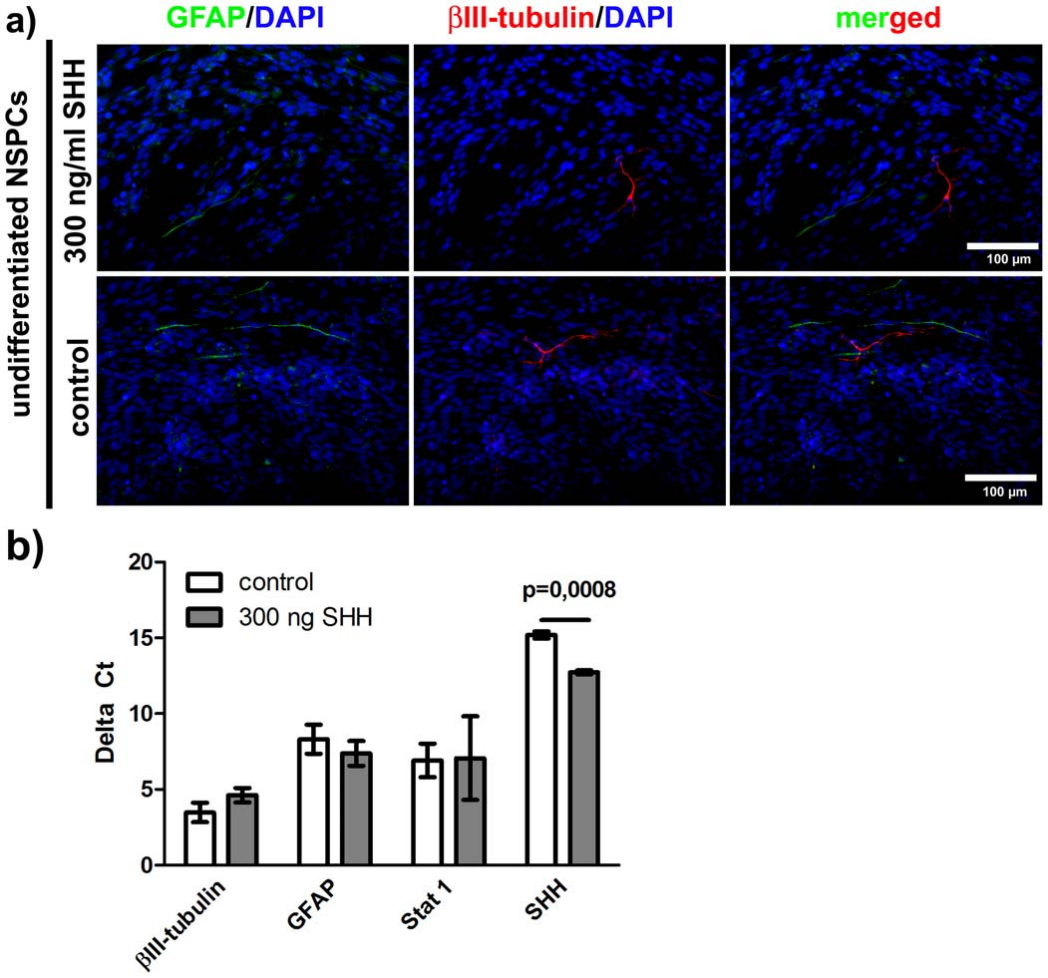
The administration of recombinant SHH does not lead to a dysregulated phenotype in NSPCs. In Figure 3a: Immunocytochemical photomicrographs of undifferentiated murine NSPCs are presented under the illustrated conditions. In Figure 3b: The results of real-time quantitative PCR are depicted. The values represent delta CT values. Delta CT values are means +/− standard error of mean (SEM). Independent experiments were repeated for three times in triplicate.

As SHH antagonism diminished the generation of IFNγ-induced GFAP^+^/βIII-tubulin^+^ phenotypes, we investigated if SHH alone is sufficient to induce this phenotype which would implicate a linear down-stream signaling IFNγ – SHH – GFAP^+^/βIII-tubulin^+^ phenotype. For this purpose, we applied 300 ng/ml recombinant murine SHH to undifferentiated NSPCs under the influence of growth factors. However, neither an increase in numbers of GFAP^+^/βIII-tubulin^-^ or GFAP^−/^βIII-tubulin^+^ cells nor the appearance of dysregulated GFAP^+^/βIII-tubulin^+^ cells was detectable. These findings were confirmed by immunocytochemistry and on gene expression level by real-time quantitative PCR (Figure 3a+b). Also, no alteration in Stat 1 gene-expression was detectable after SHH treatment. Instead, we found SHH gene-expression to be significantly up-regulated after administration of recombinant SHH to undifferentiated NSPCs implying a positive autoregulatory loop. These results suggest, that the dysregulated phenotype of NSPCs is not inducible by SHH alone, even when applied at higher concentrations.

### Stat 1 Interaction with the SHH Promoter

**Figure 4:**
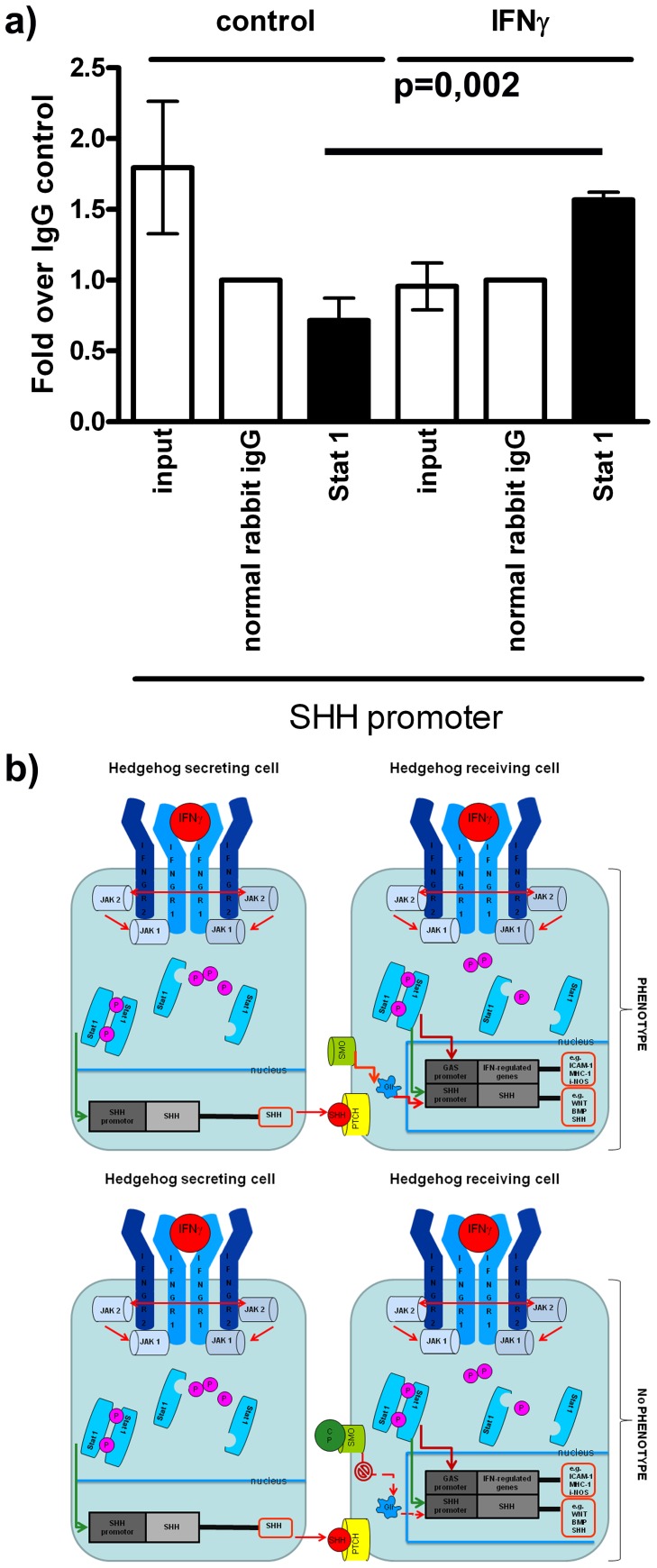
The mechanism of IFN γ**-mediated dysregulation of NSPCs.** In Figure 4a: The results of the chromatin immunoprecipitation of the SHH promoter region with a Stat 1 antibody are shown. In Figure 4b: A schematic drawing of potential pathways that might be involved in IFNγ-induced NSPC dysregulation. CP: Cyclopamine, SMO: smoothened, PTCH: patched, GAS: interferon-*gamma*-*activated site* (GAS), ICAM-1: intercellular adhesion molecule 1, MHC-1: major histocompatibility complex 1, i-NOS: inducible nitric oxide synthase, JAK2: janus kinase 2, Stat 1: signal transducers and activators of transcription 1.

To further investigate the up-regulation of SHH after IFNγ treatment and to clarify if Stat 1 binds to an IFNγ-activated side (GAS) sequence in the promoter region of the SHH promoter, we performed chromatin immunoprecipitation. For this purpose NSPCs were treated with IFNγ or cultured under control conditions and afterwards chromatin immunoprecipitation (with Stat 1 antibody, normal rabbit IgG antibody or Histon H3 antibody as positive control) was performed. After immunoprecipitation, real-time quantitative PCR against a GAS Sequence in the SHH promoter and a reference gene sequence was used to analyze possible binding of Stat 1 in the SHH promoter region. We chose primer sequences that were specifically designed to amplify GAS sequences in the SHH promoter [7]. We could show a 1.5 fold increase in the chromatin immunoprecipitation sample of IFNγ treated cells for the SHH promoter. Our findings suggest, that a phosphorylated dimer of Stat 1 is able to enter the nucleus, and then binds to a GAS sequence in the SHH promoter (Figure 4a) and therefore leads to the up-regulation of SHH after IFNγ treatment. A possible mechanism for this interaction is visualized in Figure 4b.

## Discussion

In the last few years various studies were aimed at elucidating the role of the pro- inflammatory cytokine IFNγ during neurodegenerative or neuroinflammatory conditions and its impact on NSPCs. In those reports Janus-faced properties of IFNγ were described. On the one hand, pro neurogenic effects of IFNγ on neural stem cell differentiation were described [10], [11], [12], [13], [14]. On the other, a number of publications report a negative effect on neuronal differentiation and neurite outgrowth [15], [16], [17], [18], [19], [20], [21]. A dualistic role of IFNγ was also seen when proliferation and differentiation of astrocytes was investigated [5], [22], [23].

We recently reported that IFNγ leads to phenotypic and genotypic dysregulation in a substantial portion of murine E14 neurosphere-derived stem/precursor cells. This dysregulation is characterized by a simultaneous expression of neuronal and glial markers in immature NSPCs as soon as 3 to 6 hours after application of IFNγ. Furthermore, it was shown that IFNγ causes an up-regulation of SHH and Stat 1 mRNA and a decrease in the population size.

We now extended those investigations and found that the IFNγ-induced decrease in the population size is concentration dependent beginning with 100 Units per ml. Although there might be a bias towards lower population sizes in differentiated cultures that bear a lower metabolic activity, the IFNγ-induced reduced values in the MTT assay are best explained by anti-proliferative and/or pro-apoptotic effects. This is substantiated by the observation that the IFNγ-induced dysregulation was accompanied by an up-regulation of caspase 3/7 and a decrease in BrdU uptake, suggesting IFNγ-related anti-proliferative effects and enhanced apoptosis [6].

We found that also the IFNγ-induced up-regulation of SHH and Stat 1 is concentration dependent beginning with 100 Units per ml. We speculated that SHH, as one of the major morphogenes in brain development, is involved in the formation of GFAP^+^/βIII-tubulin^+^ cells and that Stat 1 might be involved in SHH up-regulation. To substantiate this hypothesis, we first investigated if Stat 1 can bind to the SHH promoter. For this, we performed a chromatin immunoprecipitation assay and confirmed that a phosphorylated dimer of Stat 1 is capable to enter the nucleus in order to bind to a GAS sequence in the SHH promoter region and therefore leads to an up-regulation of SHH. This shows that IFNγ-related Stat 1 up-regulation is directly linked to the induced SHH pathway since its known, that the phosphorylated dimers of Stat 1 enter the nucleus and function as transcription factors [24], [25], [26].

To investigate if an activated SHH pathway is directly involved in generating the IFNγ-induced phenotype, we antagonized the SHH pathway by administration of cyclopamine. Cyclopamine is known to inhibit the activity and down-stream-signaling of smoothened by a binding-induced conformational change in the smoothened protein [9]. We were able to show that the IFNγ-induced dysregulation of NSPCs is significantly ameliorated after antagonizing SHH signaling indicating that SHH signaling is necessary to induce the dysregulated phenotype. This finding was confirmed by immunocytochemistry and real-time quantitative PCR. Notably, we were not able to completely block IFNγ-induced NSPC dysregulation by cyclopamine. This could probably be due to the low concentration of cyclopamine as it was used in our study to prevent toxicity. After identification of SHH as mediator for NSPC dysregulation, we added recombinant murine SHH in high doses to undifferentiated NSPCs, to clarify if the dysregulated phenotype can be induced solely via SHH signaling downstream from IFNγ. However, SHH alone was not able to induce NSPC dysregulation. This indicates that the IFNγ-induced dysregulated phenotype is depending on activation of the SHH pathway and simultaneously on other IFNγ-related effects. Thus, the dysregulated phenotype could either be induced via a simultaneous SHH- and Stat 1-activation of the SHH promoter or, for instance, via simultaneous SHH-activation of the SHH promoter and activation of common [27], [28], [29] IFN-regulated genes (IRGs) or IFN-stimulated genes (ISGs) by the JAK/Stat cytokine pathway. We summarized this in the schematic drawing. The IFNγ-activated site (GAS) element in the SHH promoter region might therefore be involved in SHH up-regulation or in a direct co-activation of the dysregulated phenotype.

The binding of phosphorylated Stat 1 to the SHH promoter after IFNγ exposure was previously shown by Sun et al. [7]. Interestingly, Sun and colleagues did not find a dysregulated phenotype or a reduced proliferation, but they described a clear up-regulation of SHH after IFNγ administration and they also describe a binding of Stat 1 to the SHH-promoter region. Notably, the cells used by Sun and colleagues were granular neural precursor cells from postnatal mice, in contrast to NSPCs or the murine ES cell-derived neural stem cells used in our study. This suggests that effects mediated by IFNγ exposure seem not only to depend on the examined brain region but also on the developmental stage and the cell type investigated. It is even more surprising that Todoric and colleagues described a cross-talk of IFNγ and SHH in human and murine pre-adipocytes as well [8]. This leads to the hypothesis, that crosstalk of IFNγ and SHH is an important genetically conserved factor in cellular response to inflammatory signals, since this pathway is not limited to the murine species or a special cell type. Further experiments to elucidate this hypothesis could be I) the application of JAK inhibitors, to block IFNγ signaling to the nucleus and studying the SHH expression afterwards, II) the administration of exogenous SHH together with IFNγ, to see if the amount of dysregulated cells increases by exogenously applied SHH, as well as III) a Stat 1 phosphorylation assay to measure the increase of phosphorylated Stat 1 molecules in a dependency to the used Units of IFNγ. This experiment would further confirm a direct receptor mediated phosphorylation of Stat 1 in NSPCs.

Taken together these findings provide new evidence for the importance of pro-inflammatory signals in cell fade- and differentiation-decisions since SHH is an important morphogene in brain development and the neural stem cells niche [30].

Therefore, we claim a complex and diverse role of IFNγ as mediator of dysregulation in NSPCs.

## Materials and Methods

### Neurosphere Cultures

Neurospheres were generated from fourteen-day-old wild type C57BL/6J mouse embryos. Cell preparation and animal care were performed in compliance with the German Animal Protection law (State Office, Environmental and Consumer Protection of North Rhine-Westphalia). Ganglionic eminences were removed, mechanically dissociated and seeded in DMEM/F12 culture medium (1∶1; Invitrogen, Karlsruhe, Germany) containing 0.6% Glucose (Sigma-Aldrich, Hamburg, Germany), glutamine (2 mM; Invitrogen), sodium bicarbonate (3 mM; Invitrogen), Hepes buffer (5 mM; Invitrogen) and B27 (20 µl per ml; Invitrogen). For generation and expansion of neurosphere cells, epidermal growth factor (EGF) (Tebu-bio, Le Perray en Yvelines Cedex, France) and basic fibroblast growth factor-2 (FGF-2) (Tebu-bio) were added to a final concentration of 20 ng per ml each.

### IFNγ Treatment and Immunocytochemistry

For immunocytochemistry, neurosphere cells were dissociated to a single cell suspension and plated on poly-L-ornithine (PLO; 0.001%; Sigma-Aldrich) and fibronectin (5 µg/ml; Tebu-bio) coated cover slips (VWR International, Darmstadt, Germany) at a density of 125,000 cells per cm^2^. After 3 days under the influence of EGF and FGF-2 (20 ng/ml both Tebu-bio), cells were assigned to the different experimental groups. To verify the marker expression of undifferentiated (proliferating) neural populations under control or IFNγ treated conditions, cultures were kept under the influence of EGF/FGF-2 without or with IFNγ (1000 U/ml; Millipore) until fixation for further 3 days (NSPC-p -IFNγ/+IFNγ). For control experiments, only phosphate-buffered saline solution (PBS; 1X; Invitrogen) was added to the medium. Primary antibodies used at 4°C overnight were the monoclonal mouse antibody against βIII-tubulin (Tuj1; 1∶500; R&D Systems, Minneapolis, USA or 1∶800, Abcam, Cambridge, UK) and the polyclonal rabbit antibody against glial fibrillaric acid protein (GFAP) (1∶500; Dako, Hamburg, Germany or 1∶1000; Abcam). For detection of primary antibodies, fluoresceine-isothiocyanate (FITC; 1∶500; Millipore) and indocarbocyanine (Cy3; 1∶800; or Cy5; 1∶200; Millipore) coupled secondary antibodies were used. For negative controls, primary antibodies were omitted in each experiment. To measure the total population of cells, Dapi positive cell nuclei were counted. On every cover slip, at least 100 cells were counted.

### MTT-Assay

To analyze the population size of NSPCs, the optical density, indicative of conversion of 3-(4, 5-dimethylthiazol-2-yl)-2, 5-diphenyltetrazolium bromide (MTT; Sigma-Aldrich) into formazan crystals which takes place in live cells only, was determined after IFNγ treatment at indicated concentrations. An OD value of 0.5 represents approximately 50,000, and an OD value of 1.0 represents approximately 100,000 live NSPCs. The population size was measured after 72 hours of IFNγ treatment. Therefore cells were incubated with MTT (final concentration 0.5 mg/ml) and media one half each and incubated for 3–5 hours at 37°C afterwards the supernatant was discarded and a lysis with DMSO was performed.

### Real-time Quantitative PCR

RNeasy Kit (Qiagen) was used for RNA isolation of cultured NSPCs. Then a reverse transcription into cDNA (ABI, Darmstadt, Germany) was performed. Real-time quantitative PCR was carried out by the usage of the 7500 fast or 7500 real-time quantitative PCR cycler (ABI, Darmstadt, Germany). Either SYBR green master mix (Qiagen) or equivalent chemistry from another supplier (Quantace, London, UK) was used. The specific primers for genes of interest or the housekeeping gene (glyceraldehyde- 3-phosphate dehydrogenase, GAPDH) were either purchased (QuantiTect primer assays, Qiagen) or self designed (BioTEZ, Berlin, Germany). The genes of interest (target gene) in IFNγ-treated groups or control groups (PBS-treated) were analyzed in at least 3 independent cultures in triplicate each. Every experiment in IFNγ-treated or control (PBS-treated) groups provided delta CT values (ΔCT: gene of interest minus reference gene). NSPCs were cultivated for 72 hours with IFNγ at the indicated concentrations before the RNA was harvested.

### Chromatin Immunoprecipitation Assay

Chromatin immunoprecipitation (CHIP-Assay) was performed by using the simpeCHIP IP Kit (cell signaling) according to the manufacturers instruction, with chip grade antibodies against Stat 1 (cell signaling). Briefly, NSPCs were seeded on PDL and fibronectin coated dished and treated with IFNγ or PBS as control for three days and then fixed with 1% PFA for 10 minutes, harvested and lysed. Chromatin was digested by sonification and incubation with micrococcal nuclease. For chromatin immunoprecipitation the isolated chromatin was incubated with normal rabbit IgG antibody as negative control, Histon H3 antibody as positive control and Stat 1 antibody over night at 4°C. Afterwards the chromatin/antibody mixture was incubated with protein G agarose beads for 2 hours at 4°C. The chromatin was washed and eluted from the protein g agarose beads, the DNA was reverse cross linked to single strand formation and purified over silica membranes. After purification, the isolated DNA was used for real-time quantitative PCR using primers against the SHH promoter regions and the histon H3 complex.

### Statistical Analyses

Experiments were repeated with independent cultures at least three times in triplicate each. The resulting data sets were statistically analyzed und illustrated using the GraphPad Prism 4 (GraphPad Software Inc., San Diego,CA, USA, 2003) software. For approval of statistical significance between groups, a two-tailed unpaired t-test was performed. P values <0.05 were considered to indicate significant differences.
